# Paddynet: An organized dataset of paddy leaves for a smart fertilizer recommendation system

**DOI:** 10.1016/j.dib.2023.109516

**Published:** 2023-08-23

**Authors:** Md. Moradul Siddique, Torikul Islam, Yeasir Arefin Tusher, Romana Rahman Ema, Md. Nasim Adnan, Syed Md. Galib

**Affiliations:** a Department of Computer Science and Engineering, Jashore University of Science and Technology, Jashore 7408, Bangladesh; b Institute of Information Technology, University of Dhaka, Dhaka 1217, Bangladesh

**Keywords:** PaddyNet, Nitrogen recommendation, Leaf color chart, LCC color recognition, LCC dataset, LCC automation, Paddy leaves, Fertilizer recommendation system

## Abstract

The dataset of Leaf Color Chart (PaddyNet) is publicly unavailable. As far as the author's knowledge, this is the first dataset about paddy leaves based on LCC. This dataset has been generated by collecting images from a particular location such as Sajiali, Dogachia and Shyamnagar at Jashore, Bangladesh. This dataset contains 4 categories of Aman paddy leaves. The leaf images were captured by smart phones. There are 560 images of Aman paddy leaves. The data collection procedure was carried out according to the guidelines of Bangladesh Agricultural Research Institute (BARI). We meticulously categorized the entire dataset with regard to the LCC level and validated the data with the assistance of domain specialists. Hence, the images are analyzed and categorized with standards. The dataset is utilized for recognizing Leaf Color Chart level which will help of farmers recommending nitrogen fertilizer in their paddy fields.

Specifications Table

**Table:** 

Subject	Pattern Recognition and Computer Vision
Specific subject area	Recognizing Leaf Color Chart (LCC) level
Type of data	Image
How the data were acquired	For data collection, we have developed an android application and used Nokia 3 (8 MP camera) and Samsung S8 (12 MP camera) smartphones. The captured images were obtained at 720p@30fps and 720p@240fps resolution, respectively. After data collection, the images are subjected to filtering, labeling, semantic segmentation, and augmentation prior to being reduced to 500×500 pixels.
Data format	PNG JPG Filtered Segmented Augmented Analyzed
Description of data collection	All the 560 images have been categorized into four distinct levels. The levels have been assigned numbers such as 2, 3, 4, and 5; - where each of the two to five LCC levels includes 158, 162, 124, and 116 images, respectively. The images are focused on color of paddy leaves with RGB format.
Data source location	Location: Sajiali, Dogachia and Shyamnagar, Zone: Jashore sadar, Khulna, Bangladesh.
Data accessibility	**Repository name:** Mendeley Data **Data identification number:** DOI:10.17632/ksz57tk5vc.2 **Direct URL to data:** Siddique, Md Moradul; Islam, Torikul; Tusher, Yeasir Arefin; Md. Galib, Syed (2023), “PaddyNet: An Organized Dataset of Paddy Leaves for a Smart Fertilizer Recommendation System ”, Mendeley Data, V2, doi:10.17632/ksz57tk5vc.2[1]
Related research article	Islam, Torikul, Rafsan Uddin Beg Rizan, Yeasir Arefin Tusher, Md Shafiuzzaman, Md Alam Hossain, and Syed Galib. ``Nitrogen fertilizer recommendation for paddies through automating the Leaf Color Chart (LCC).'' *International Journal of Advanced Computer Science and Applications* 11, no. 8 (2020). https://doi.org/10.14569/IJACSA.2020.0110891

## 1 Value of the Data

• PaddyNet, a comprehensive dataset, comprises 6000 images of paddy leaf that can classify the color of paddy leaf with human eyes. As an outcome, the researchers can effectively contribute to data analysis and identify the level of paddy leaf.
• The level of paddy leaf images can be identified by classifying, comparing, testing, and estimating data using various machine and deep learning-based attribute selection methods.
• The dataset can be utilized to develop advanced paddy images categorization and detection based on a leaf color chart.
• The dataset can be used to develop an application for paddy leaf color identification that will assist farmers to estimate fertilizers in paddy fields.
• PaddyNet will significantly contribute to the elimination of manual barriers with the Leaf Color Chart.

## 2 Objective

The primary goal of generating the PaddyNet dataset is to support researchers in developing a framework that helps farmers overcome their manual barrier. The dataset will substantially assist researchers in conducting research on nitrogen fertilizer recommendations in paddy fields. We have developed a unique dataset which is the first Leaf Color Chart (LCC) dataset according to knowledge of Bangladesh Agricultural Research Institute (BARI), Jashore, Bangladesh. This dataset has been employed to support an already published original research study. Consequently, the data contributes to the value of the published research paper by clearly presenting functionality of the data as well as its efficiency in recognizing the level of Leaf Color Chart. The article will provide readers with a comprehensive understanding of the PaddyNet dataset. This article will contribute to easily understand the data and utilize the dataset as well as conduct an additional study on fertilizer recommendation of paddy fields.

## 3 Data Description

**Fig. 1:**
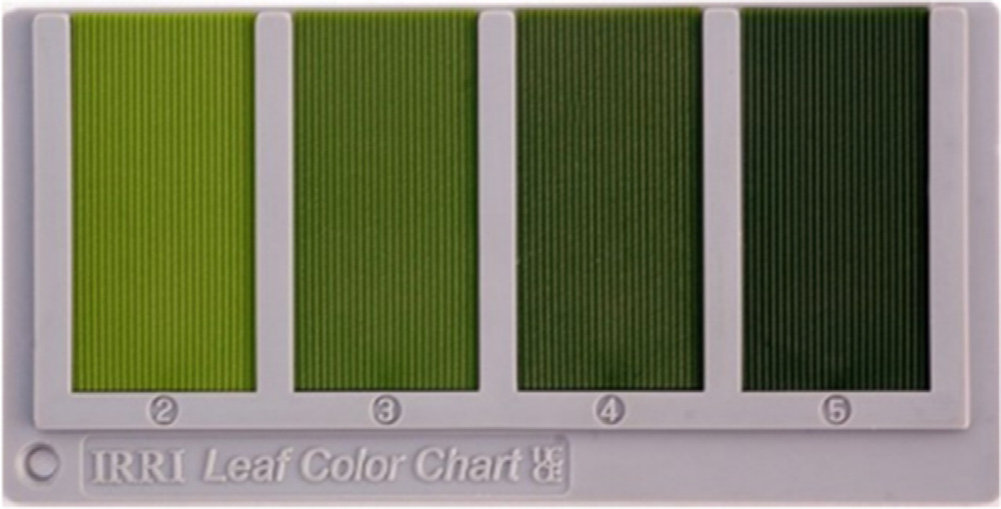
Leaf Color Chart (LCC) [6]. (For interpretation of the references to color in this figure legend, the reader is referred to the web version of this article.)

According to International Rice Research Institute (IRRI), Leaf Color Charts (LCC) - shown in Fig. 1 - can help farmers determine an approximation of the nitrogen level of paddies through matching the color of paddy leaves. Thus, automating the LCC process will be a help to the introduction of smart farming in Bangladesh. Farmers can get fertilizer recommendations through automated LCC easily. In this way, overestimation or underestimation may be avoided through smart farming [2]. So far, there is no Leaf Color Chart dataset available publicly. In other context, similar experiments on nitrogen fertilizers recommendation for soybean, a Fuzzy Logic technique was developed [3]. Mercado-Luna et al. [4] assessed the nitrogen requirements of tomato seedlings through image analysis. With the help of a chlorophyll meter and an LCC, sing et al. [5] calculated the nitrogen utilization based on requirements for wheat and rice.

In this study, we have generated a Leaf Color Chart (PaddyNet) dataset for recognizing 4 useful color level of paddy leaves. Therefore, our aim was to collect paddy leaf images and label them by categorizing them into 4 different levels. Hereby, LCC exists in the ranges from 2 (yellowish green) to 5 (dark green), which is exhibited in Fig. 1. Our presented dataset can serve as a state-of-the-art reference for building color recognition algorithms for paddy leaves. Our currently collected dataset consists of 560 original images and 6000 augmented paddy leaf images. In this experiment, augmented paddy leaf images saved in the jpg file format. Table 1 outlines the image distribution that the dataset contains in its entirety.

**Table 1:** Distribution of dataset by class.

Class name	Quantity of original images	Quantity of augmented images
Level 2	158	1500
Level 3	162	1500
Level 4	124	1500
Level 5	116	1500
Total	560	6000

Our team has collected paddy leaf images according to the instructions of a plant expert from the Bangabandhu Sheik Mujib Agriculture University (BSMRAU), Gazipur. As a response, Paddy images were snapped in the daytime within the body shadow. The paddy leaves were held in one hand and within six to eight inches distance, the images were captured. Over a period of 70 days, the images were captured in a wide range of lighting and atmospheric situations. From the middle of April to the end of June, we collected a significant number of images of paddy leaves.

**Fig. 2:**
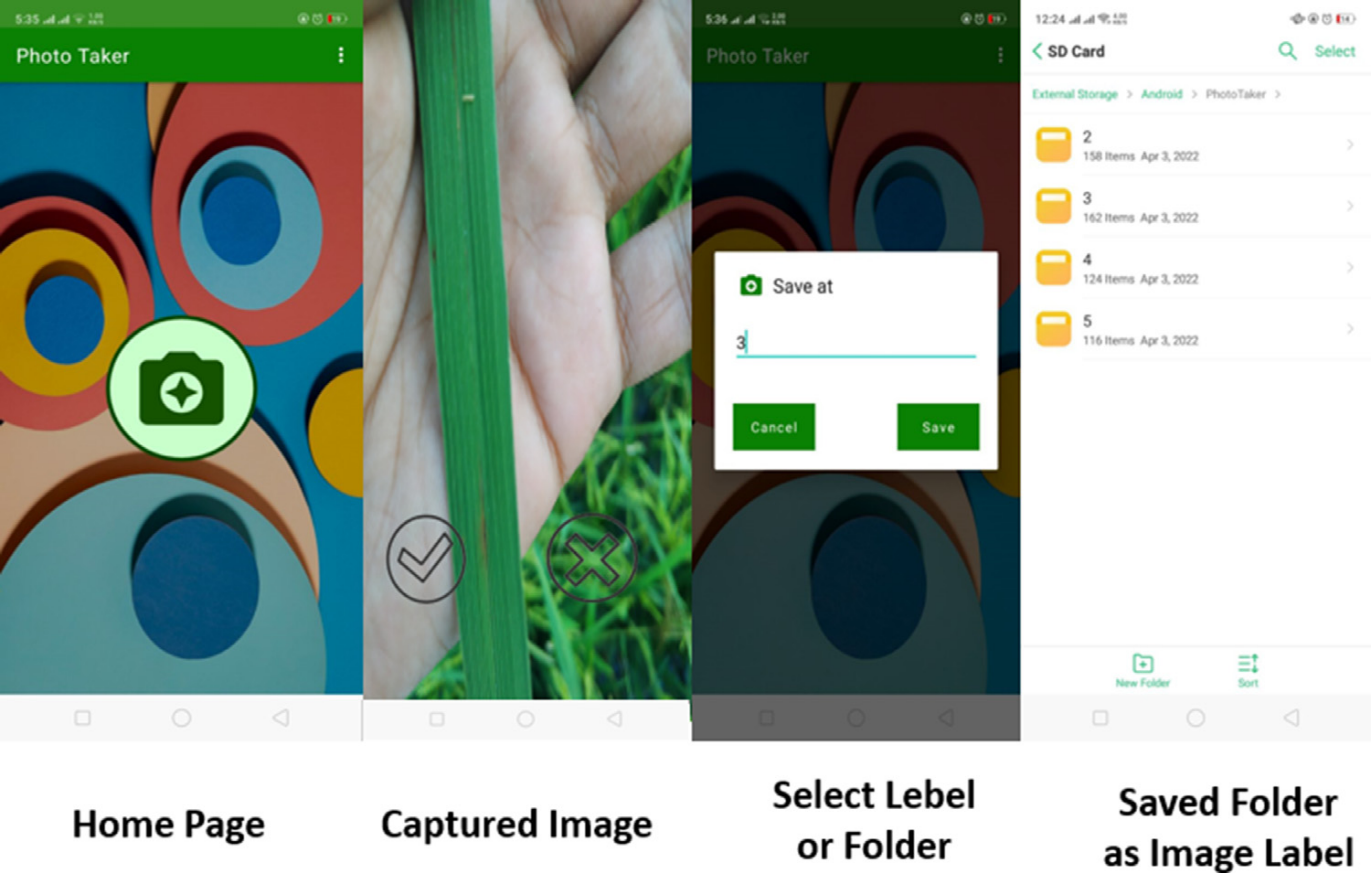
Implemented an image dataset acquisition application. (For interpretation of the references to color in this figure legend, the reader is referred to the web version of this article.)

**Fig. 3:**
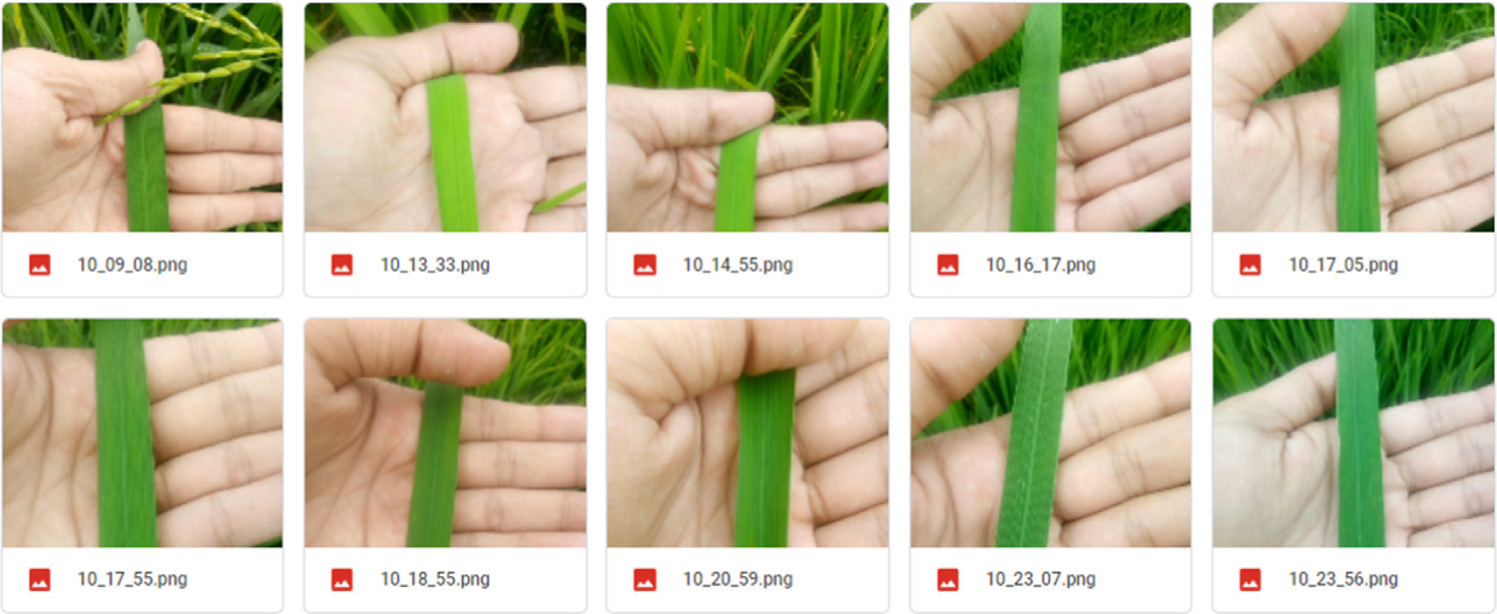
Sample images of paddy leaves taken from the field. (For interpretation of the references to color in this figure legend, the reader is referred to the web version of this article.)

The collection of data plays a vital part in preventing the waste of time and money. Data collection via mobile phone is a fast and efficient method. For this reason, we have made an android application to easily collect the images. We have collected data using our Photo Taker application on Nokia 3 (8 MP camera) and Samsung S8 (12 MP camera) devices. In Fig. 2, we demonstrate the data collection procedure used in the application. Each of the image samples was finally saved in the png file format. The sample images of paddy leaves taken from the field is presented in Fig. 3.

## 4 Experimental Design, Materials and Methods

**Fig. 4:**
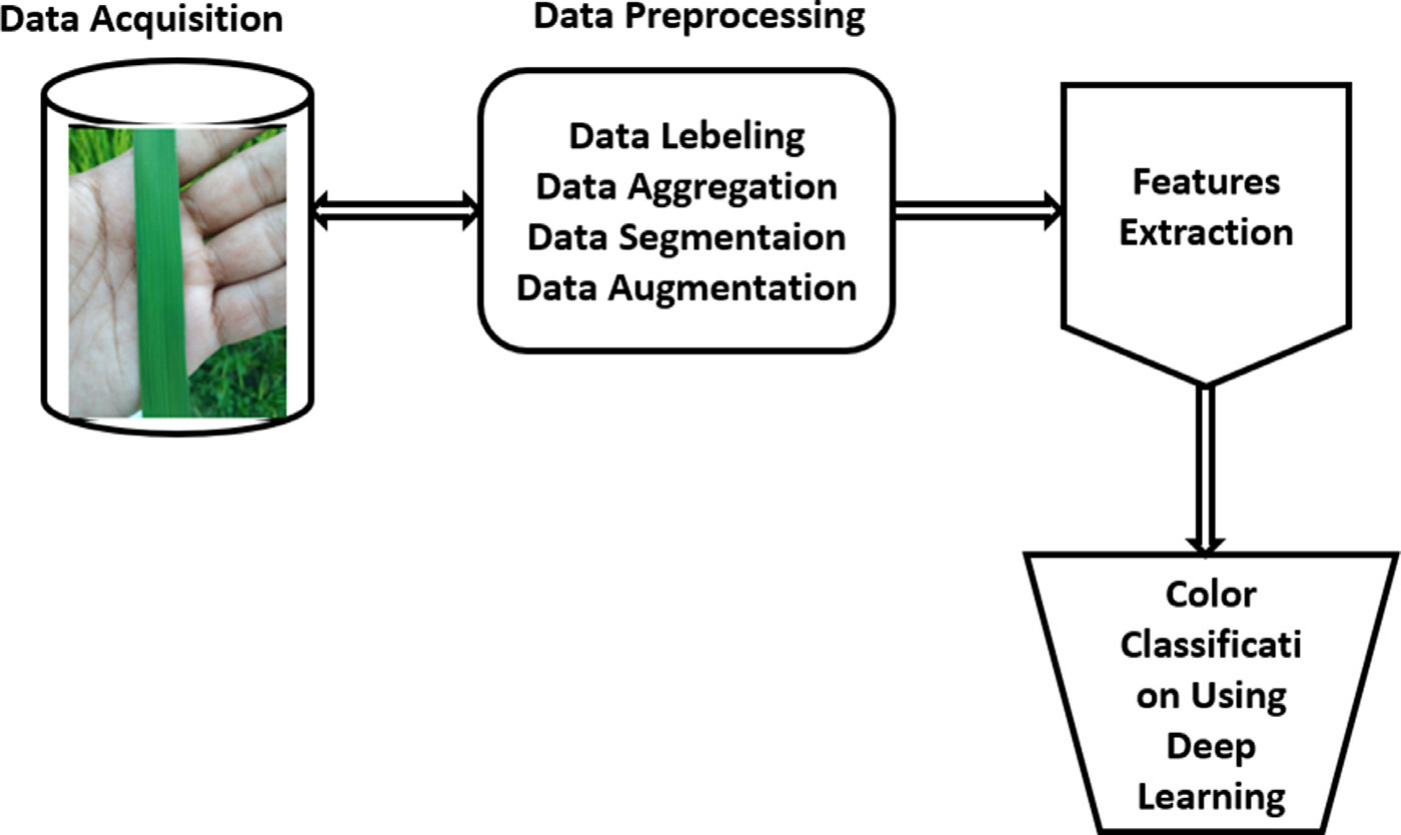
Traditional Approach of Leaf Color Recognition from the Images. (For interpretation of the references to color in this figure legend, the reader is referred to the web version of this article.)

**Fig. 5:**
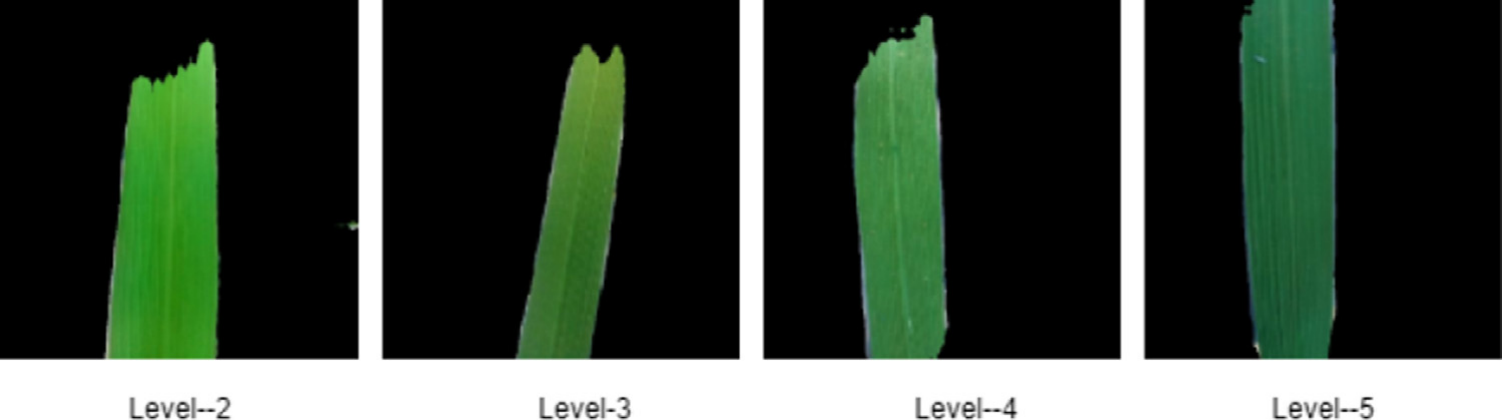
After processing, sample images of LCC four level [7]. (For interpretation of the references to color in this figure legend, the reader is referred to the web version of this article.)

The conventional process of detecting the leaf color based on the images is depicted in Fig. 4. According to LCC, the process of getting different shades of color from leaves is referred to as image acquisition. The identification process of leaf color includes two parts, such as image preprocessing and model development (ML and DL). The images were taken from several agricultural field. These images are treated further by applying image processing techniques. Finally, deep learning approaches classify the color level based on image attributes. Background removal, noise reduction, resizing, labeling, aggregation, augmentation, and segmentation are all instances of image processing stages which are depicted in Fig. 5, whereas feature selection and classification are instances of deep learning. Also, the classification has been classified using a ML classifier named Decision Tree (DT).

## 5 Data Preprocessing

After analyzing and organizing the dataset, before analyzing the data, acquired data had to be cleaned and preprocessed. Background of the collected images were removed, leaf images were isolated using segmentation, images were augmented so that any orientation of images can be recognized and also the number of data is increased for training purpose. Finally, the images were labeled according to expert opinion.

## 6 Data Segmentation

DeepLabv3+ is a highly effective model for semantic segmentation which is a computer vision task that involves assigning a class label to every pixel in an image. It encourages the network to learn meaningful representations at various resolutions, leading to improved performance. Undoubtedly, it is a crucial component of computer vision is the process of semantic segmentation with the goal of providing a semantic label to every single pixel in the frame. Owing to do multi-class semantic segmentation, we employ the DeepLabV3+ model [8], a fully-convolutional architecture that has shown promising results on semantic segmentation benchmarks.

## 7 Data Augmentation

A technique called image data augmentation produces modified replicas of the images that are present in the training dataset. Due to this, the dataset's size can be artificially extended without the requirement for additional data collection. More data is better when it comes to training models for deep neural networks, which can lead to more accurate predictions [9].

**Fig. 6:**
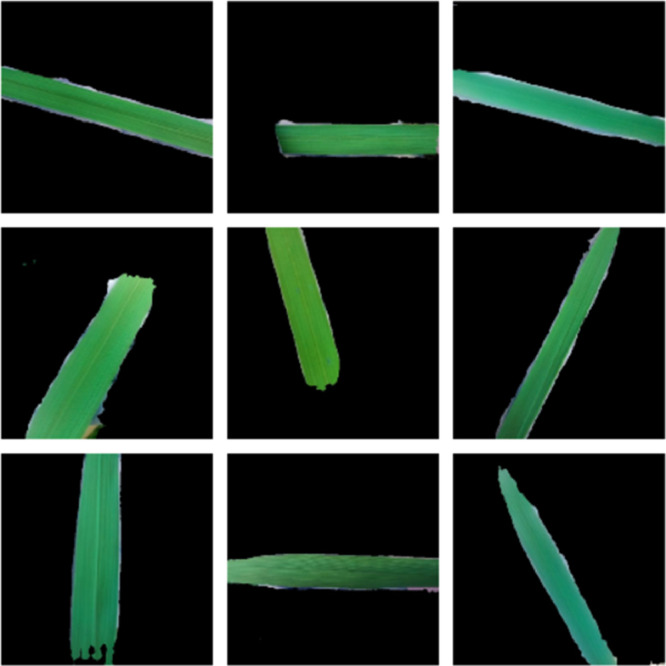
A sample of augmentation on PaddyNet dataset. (For interpretation of the references to color in this figure legend, the reader is referred to the web version of this article.)

In order to help fit models, generalize the findings to new images, augmentation methods might generate variants of the original input images. In order to improve upon our initial 560 paddy leaf images, we have increased the number to 6000, with 1500 images for each LCC level. Color pixel data is preserved by the combination of augmentation factors such as shear, width and height shift, zoom and horizontal flip [10] that is shown in Fig. 6.

## 8 Classification Methods

**Fig. 7:**
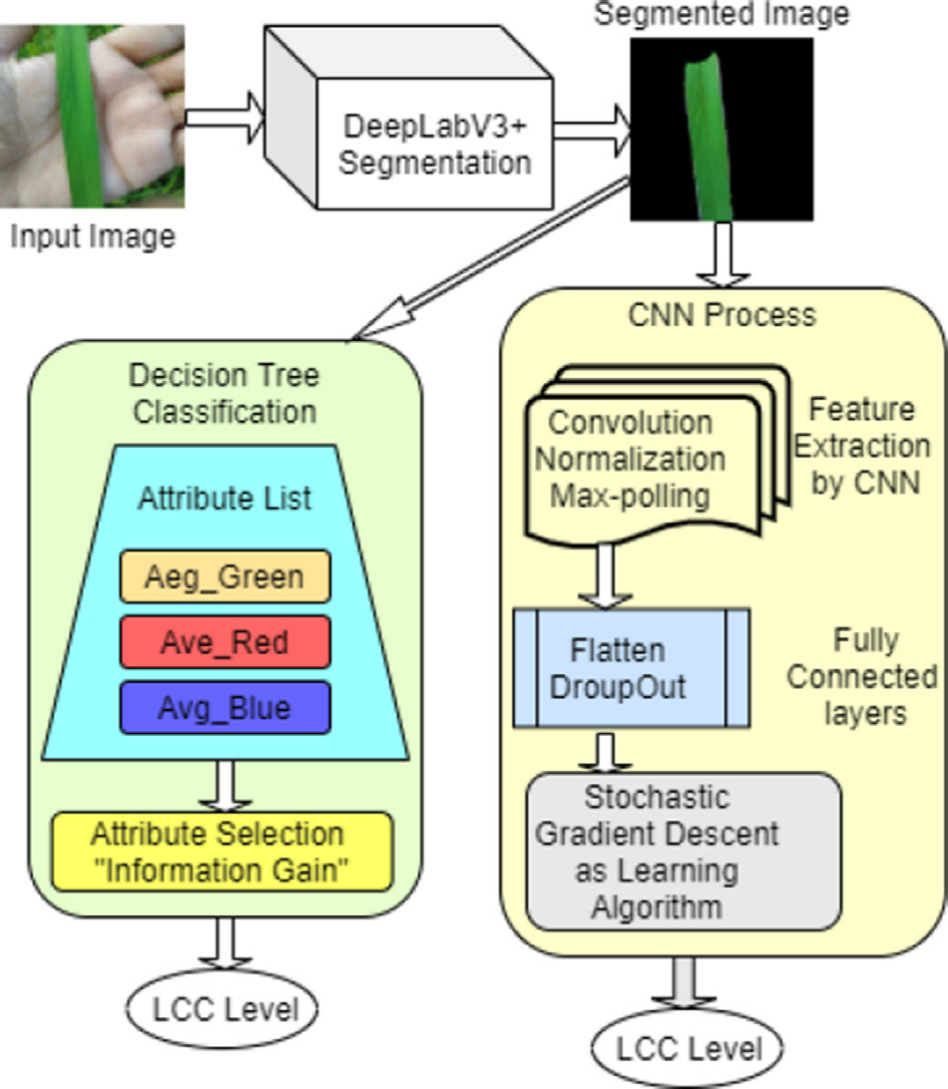
Workflow of proposed two methods [7]. (For interpretation of the references to color in this figure legend, the reader is referred to the web version of this article.)

Our entire data set of 6000 images has been divided into 70% of training data and 30% of testing data. The images were split into train and test after augmentation. The dataset is evaluated by Deep Learning model particularly Convolutional Neural Network (CNN) technique to recognize the 4 levels of LCC. Besides, we have used the most popular machine learning techniques is Decision Tree. The workflows of the proposed two methods are illustrated in Fig. 7. While training our dataset for evaluation, the models have achieved 94.22% and 91.22% accuracy for CNN and DT respectively. The result shows that the Deep Learning model (CNN) achieves better accuracy than Machine Learning model (DT). Furthermore, on training data, cross-validation was not employed. However, we have tested the model's performance on 60 completely new images, with 15 paddy images per LCC level, as part of the validation method. In this method, the DT model achieved an accuracy of 83.33% while the CNN model achieved an accuracy of 91.66%. As a result, CNN model performs better to recognize the 4 levels of paddy leaf in comparison to ML models trained on our PaddyNet dataset.

## Ethics Statements

A number of images was collected from paddy fields that comprised the PaddyNet dataset. Regarding the farmer's consent, all necessary permissions have been obtained. Additionally, hazardous or prohibited application or devices were not uses. Basically, this dataset was collected using smartphones. In addition, we have followed up several data acquisition recommendations from specialists. Procedures for the collection, storage, and processing of data are referred to as guidelines. As a result, there is no impact on paddies or paddy fields.

## CRediT authorship contribution statement

**Md. Moradul Siddique:** Methodology, Investigation, Formal analysis, Writing – original draft, Writing – review & editing. **Torikul Islam:** Methodology, Investigation, Conceptualization, Writing – original draft, Writing – review & editing. **Yeasir Arefin Tusher:** Writing – original draft, Writing – review & editing. **Romana Rahman Ema:** Conceptualization, Supervision, Investigation, Writing – review & editing. **Md. Nasim Adnan:** Supervision, Writing – review & editing. **Syed Md. Galib:** Supervision, Funding acquisition, Writing – review & editing.

## Data Availability

PaddyNet: An Organized Dataset of Paddy Leaves for a Smart Fertilizer Recommendation System (Original data) (Mendeley Data).
